# The role of pathology laboratories in integrating genetic testing into Australian primary care: an implementation science perspective

**DOI:** 10.1038/s41431-026-02213-8

**Published:** 2026-09-11

**Authors:** Maryam Vizheh, Samran Sheriff, Klay Lamprell, Romika Patel, Jeffrey Braithwaite, Janet C. Long

**Affiliations:** Australian Institute of Health Innovation, Faculty of Medicine, Health and Human Sciences, Macquarie University, Sydney, Australia

**Keywords:** Health care, Genetic services

## Abstract

As genomic testing moves into mainstream healthcare, non-genetic healthcare professionals, including general practitioners (GPs), play a critical role as gatekeepers to genetic services. Laboratories are essential in supporting this transition by providing not only high-quality genetic tests but also point-of-care tools, educational materials and clinical guidance to support their use. This study aimed to explore how these tools and supports are conceptualized, developed, implemented and evaluated and how laboratories integrate them into their relationships with GPs, an essential process for paving the way toward better use of genomics in primary care. A qualitative study design was employed using semi-structured, in-depth interviews with representatives from genetic laboratories across Australia. The Consolidated Framework for Implementation Research (CFIR) guided deductive content analysis of data. Findings spanned the four CFIR domains (Intervention Characteristics, Outer Setting, Inner Setting and Implementation Process) across 34 constructs. Participants reported that laboratories viewed point-of-care tools and resources as essential responses to persistent genomic knowledge gaps among GPs. Development of evidence-based, practice-driven and adaptable resources was supported and rewarded within laboratory organisations. A strong culture of clinical responsibility and implementation readiness, combined with robust networks and communications, enabled timely support for GPs. Gaps identified included lack of implementation planning, misalignments between laboratory-developed resources and GPs’ real-world needs and inadequate mechanisms for obtaining GPs’ feedback, which made evaluation problematic. By applying an implementation science framework, these findings provide insights for future efforts to build and sustain the provision of point-of-care tools and support, ultimately improving the integration of genomics in primary care.

## Background

As genomic testing moves into mainstream healthcare, non-genetic healthcare professionals, including family physicians or general practitioners (GPs), are increasingly playing a key role in initiating testing and follow-up for some genetic conditions [1]. Improving clinical capacity for genomics in primary care promises better health for patients, chiefly through earlier diagnosis, more targeted risk management and early intervention [2]. Nonetheless, the literature has shown that many GPs lack the necessary genetic knowledge or feel unprepared to offer genetic tests, interpret and manage results (especially in the case of high-chance carrier screening results) and communicate genetic information to patients [3, 4].

One solution is providing point-of-care tools and support for GPs in genetic testing, as there are significant differences regarding genetic information compared with other sorts of routine test results that GPs are accustomed to communicating to their patients. Clinical genetic testing is a dynamic and information-intensive field. Results have implications beyond individuals, involving family members and potential children and may also affect employment and the costs of obtaining life insurance [5]. The Australian Parliament has now passed the Treasury Laws Amendment, which prohibits insurers from using genetic test results to adversely affect an individual’s eligibility for life, income protection or trauma insurance. This law is scheduled to take effect on 8 October 2026. However, concerns remain that genetic information may still be indirectly relevant within health insurance settings, where conditions may be classified as pre-existing and subject to standard waiting periods for certain preventive or ancillary services [6, 7]. In addition, there is a level of complexity for many genetics consultations for which GPs’ training to date has been limited and requires specialised expertise [8]. In this context, confidentiality and informed consent are especially pertinent and particular care is needed in preparing patients for genetic information to ensure accurate understanding, interpretation and appreciation of the implications [9].

Pathology laboratories play an essential role at the interface between GPs and genetic testing, with their role continuing to evolve in response to increasing test complexity, expanded access and rising test ordering by non-specialist clinicians [8]. Traditionally, the roles of pathology laboratories were mainly confined to producing diagnostic reports, with minimal involvement in clinical decision-making or recommendations. Some pathology laboratories are beginning to provide more active clinical support—such as guidance on appropriate test selection, ordering processes, result interpretation and referral pathways—often facilitated through the employment of in-house genetic counselors to collaborate with physicians, for example, in Australia, the USA, Canada, etc. [10]. However, this is emerging and non-uniform rather than a routine standard of practice. These activities influence GPs’ clinical decision-making [11] and reflect a broader paradigm shift in laboratories’ roles and responsibilities.

The GP-laboratory relationship extends beyond the mere provision and interpretation of laboratory test results. GPs often maintain long-term relationships with preferred laboratories, shaped by considerations such as geographic accessibility to patients, reliability, accountability, coordination, trust, billing processes and turnaround times. At the same time, in many health systems, laboratories actively compete for GP referrals through value-added services [12]. For example, some genetics laboratories offer GPs informational resources and continuing professional development programs. Some laboratories provide patients with access to genetic counseling, either through an in-house genetic counselor or by referring to external genetic counseling services within the laboratory’s professional network [13].

These kinds of support and resources from laboratories can influence how readily genetic testing is ordered and managed, yet their implementation has not been monitored. Notably, many initiatives have developed within individual laboratories and subsequently diffused more broadly across the sector rather than through alignment with a national roadmap or formal best-practice guidance [14]. Therefore, it is crucial to examine how and why laboratories developed these kinds of support and the considerations that shaped their design, implementation and any evaluation.

In Australia, national health policy initiatives, including the routine integration of genomics into the public healthcare system through the National Health Genomics Policy Framework and Implementation Plan 2026–2030 [15], alongside research programs, have increasingly promoted the integration of genomics into primary care. However, limited data are available on how GPs receive support from pathology laboratories in ordering and interpreting the genetic tests [16]. This lack of evidence could hamper the dissemination of successful approaches to evidence translation and slow the uptake of genomics in primary care. This study aimed to explore how different Australian pathology laboratories perceive their role in supporting genomics testing in primary care by providing point-of-care tools and resources to support GPs in their daily practice.

Point-of-care tools and support (i.e., for use during or soon after a consultation) in this study refer to the concise, practical resources (e.g., flowcharts, checklists, decision aids and test-ordering templates) and the informational or clinical assistance that laboratories provide to GPs (e.g., guidance on test selection, interpretation and follow-up and the ability for GPs to seek advice directly from laboratory staff). Together, these supports are designed to help GPs navigate genomic testing efficiently and safely. We focused on point-of-care tools and support for reproductive genetic carrier screening (RGCS) (genetic variants causing cystic fibrosis, spinal muscular atrophy and/or Fragile X syndrome performed pre-conception or in early pregnancy) and testing for familial hypercholesterolaemia (FH), a common genetic variant that constrains the clearance of low-density cholesterol in the blood, predisposing individuals to premature coronary artery disease [17, 18]. The rationale for selecting these tests was that, unlike most genomic testing, which is consumer-funded or government-funded but restricted to specialist clinical genetics services, these two areas of genomics are now subsidised by Medicare (Australia’s universal health insurance scheme), increasing their affordability and pragmatic use in primary care [18, 19]. Currently, GPs are eligible to order these tests, making primary care a key point of access for eligible individuals [20].

Framing the provision of support and point-of-care tools for GPs as an ‘intervention’, the Consolidated Framework for Implementation Research (CFIR) can provide a comprehensive structure to identify and analyse the nuanced layers of providing them and organise multifaceted factors influencing their implementation in practice, while offering a taxonomy for future efforts [21, 22]. CFIR is a determinant framework that includes constructs from many implementation theories, models and frameworks. The overarching aim of the CFIR is to predict or explain barriers and facilitators (determinants, independent variables) to implementation effectiveness (the outcome, dependent variable). CFIR can thus be used to inform the choice of implementation strategies that may best address contextual determinants, generate hypotheses to prospectively guide predictions of implementation outcomes or retrospectively explain implementation outcomes by assessing differences in determinants across implementation settings [23]. In the context of this study, CFIR enables systematic analysis of how laboratory practices, structures and culture, inter-organisational networks, professional roles and policy contexts interact to shape the implementation of genomic point-of-care tools and resources in primary care settings. Using this framework, this study aimed to explore all aspects of how point-of-care tools and support are conceptualised, developed, implemented and evaluated and how laboratories integrate them into their relationships with GPs, a process that is essential for paving the way toward better use of genomics in primary care.

## Methods

A qualitative study design was used to examine the design, implementation and evaluation of point-of-care tools and resources provided by pathology laboratories to support GPs in offering and managing genetic testing in primary care.

### Eligibility criteria

Participants were eligible if they were staff members of an accredited Australian-based laboratory, including Australian divisions of global brands, offering genomic testing services, such as RGCS or FH testing. In addition, participants routinely interacted with GPs and were involved in developing, delivering or supporting point-of-care tools and resources within their scope of practice.

#### Data collection

A list of Australian-based laboratories involved in genomic testing for RGCS and/or FH testing was compiled using information from professional networks and website searches. An invitation email, including detailed information about the study and participation information sheets and consent form, was sent to laboratory representatives who were responsible for managing GP-related support, openly asking them to participate. Selection of laboratories was guided by a maximum variation approach to capture diversity in laboratory models, including public and privately funded laboratories, as well as those with and without in-house genetic counsellor support. This approach was used to ensure representation of different organisational structures and service delivery models.

There are 12 large pathology laboratory networks conducting genetic testing in Australia, mostly owned by three private groups with around 80 branches nationwide, while the remainder are operated by state or sub-state governments [24]. For the purposes of this study, sampling was conducted at the organisational/network level to capture diversity in service models across the sector. Where laboratories formed part of the same corporate group, recruitment focused on a single representative from that network to avoid duplication of highly centralised operational models and to ensure breadth across different organisations.

Online, one-on-one semi-structured interviews were conducted by MV and SS (Qualitative Researchers). All participants were required to provide written consent for the interview and return it to the researchers prior to the interview. At the beginning of each interview, verbal consent was also sought to record the interview. All interviews were conducted using Zoom, Version 6.5.11, which enabled video recording and automated transcript generation. Each transcript was subsequently reviewed and proofread for accuracy by MV. Before commencing data analysis, all transcripts were de-identified to maintain participant confidentiality. Each interview lasted approximately 30–40 min. Each interview started with some information about the laboratory’s scope and the different kinds of genetic tests they perform. Then, some specific questions were asked to assess the point-of-care tools and support provided by laboratories. Some of the questions included in the interview schedule were:

- How do you view the role of genomics laboratories in supporting general practice in Australia?
- What common challenges or queries do GPs raise about ordering or interpreting genetic tests? How do you address this feedback?
- Have you developed or do you consider it within your scope to develop, clinical decision support tools (e.g., point-of-care prompts, risk assessment tools) that integrate into GP software?
- How do you ensure these resources are evidence-based, ethically sound and tailored to diverse populations?

The full interview schedule is provided in Supplementary File 1.

### Data analysis

An implementation science-informed approach was used to analyse data. The CFIR involves 39 constructs across five domains (Intervention Characteristics; Outer Setting; Inner Setting; Characteristics of Individuals; and Process) [21]. MV developed a coding guide derived from the CFIR domains and constructs with context-specific definitions and then analyzed transcripts using deductive content analysis [25]. Results were presented to the wider team and when the choice of construct or domain was questioned, it was discussed with team members until consensus was reached. The original definitions of CFIR domains and constructs, along with adapted definitions applied in the context of this study, are presented in Supplementary File 2. Data were managed in NVivo 12.

## Results

### Participants’ characteristics

A total of 14 representatives from 12 pathology laboratories conducting genetic testing across Australia were identified and approached. Of these, representatives from eight laboratories agreed to participate and all were included in the study. These laboratories collectively provide coverage across all eight Australian states and territories, encompassing both metropolitan and regional areas. Table 1 shows the geographic coverage of these laboratories. In all participating laboratories, genetic testing for selected conditions (FH and RGCS) was covered by Medicare.

**Table 1:** The geographic coverage of pathology laboratories represented in this study.

Laboratory number	Role/ Area of expertise of interviewees	Coverage	Privately or publicly funded
LR*1	Deputy Director of Genetics, Genetic Pathologist	National (all states & territories)	Private
LR 2	General Manager	Australian Capital Territory & surrounding region	Public
LR 3	Senior Scientist	National (all states & territories)	Private
LR 4	General Manager	National (all states & territories)	Private
LR 5	Head of Service Delivery and Development: Clinical Genomics	Victoria & some regional outreach	Public
LR 6	Genetic Pathologist	National (all states & territories)	Private
LR 7	Senior Hospital Scientist, Molecular Genetics	New South Wales	Public
LR 8	Senior Hospital Scientist, Molecular Genetics	New South Wales	Public

^*^LR Laboratory representative.

### Point-of-care tools and supports currently provided by pathology laboratories

A summary of the current point-of-care tools and support to assist GPs in ordering and managing genetic tests, as reported by the pathology laboratories, is presented in Table 2. These data reflect laboratory-reported availability and provision of tools and supports for GPs. Actual utilisation by GPs was not independently verified in this study. While varied across different laboratories, the most common form of support was post-result counselling for patients, particularly in the case of high-chance results, referring to results indicating an increased probability of an affected pregnancy based on inheritance patterns [26].

**Table 2:** Point-of-care tools and supports are reported by pathology laboratories as being available now to clinicians.

Laboratory	Genetic testing offered	Point-of-care tools and supports
Laboratory 1	• Medicare covered RGCS (3-gene); • Large carrier screening panels; • Genome-wide screening • FH testing;	• In-house genetic counselling for patients and genetic recommendations for GPs with direct referral links and rapid access; • GP educational courses with Continuing Professional Development accreditation; • Guideline-based flowcharts; • Patient education modules;
Laboratory 2	• Medicare covered RGCS (3-gene); • Large carrier screening panels; • Whole genome sequencing • FH testing;	• Referral guidance via direct phone contact with laboratory; • Template-based recommendations; • Phone or/and email advice on test suitability; • Follow-up communication for complex cases;
Laboratory 3	• Medicare covered RGCS (3-gene); • FH testing; • Large carrier screening panels; • Broad range of genetic tests; • NIPT; • Inherited cancer panels; • Pharmacogenomics; • Single-gene assays;	• Linking GPs to genetic counsellors outside the laboratory for complex reports; • Providing guidance for GPs on complex test reports; • Responsive phone support lines; • Pamphlets and materials explaining tests; • Workshops and lectures for GPs on specific tests (RGCS, NIPT); • Ongoing educational sessions through GP networks
Laboratory 4	• Medicare covered RGCS (3-gene); • FH testing; • Large carrier screening panels; • NIPT;	• Clinician-facing educational materials; • Counselling Recommendation included in high-risk reports; • List of private specialists provided for GPs and patients;
Laboratory 5	• Medicare covered RGCS (3-gene); • Large carrier screening panels; • Diagnostic panels;	• Phone/email liaison service for GPs to contact laboratory to seek advice; • Clinical liaison team contacts GPs for advice on referrals, pathways and interpretation; • Web/email call centre;
Laboratory 6	• Medicare covered RGCS (3-gene); • Large carrier screening panels; • NIPT; • FH testing; • Single-gene tests (HLA-B27, celiac, Factor V Leiden, HFE)	• Post-test genetic counselling for patients with high-risk results; • Phone or/and email contact with laboratory for GPs; • Online patient-facing educational resources; • High-risk results flagged with explanatory comments in reports;
Laboratory 7	• Medicare covered RGCS (3-gene); • Large carrier screening panels; • Diagnostic genetic tests	• Recommendations through phone calls for GPs seeking advice; • Information sheets for specific tests; • Patients access genetic counselling via hospital services;
Laboratory 8	• Medicare covered RGCS (3-gene); • Large carrier screening panels; • NIPT	• Follow up with clinicians for additional clinical information; • Ad hoc support for complex results;

*GP* General practitioner, *RGCS* Reproductive genetic carrier screening, *NIPT* Non-invasive prenatal testing, *FH* Familial hypercholesterolemia.

### Thematic analysis

Four CFIR constructs were identified across the implementation of point-of-care tools and resources that laboratories reported as providing support to GPs in integrating genetic testing into primary care. The only domain not represented in our data was the ‘Individual Characteristics’ domain, which focuses on the knowledge, beliefs, self-efficacy and roles of individual staff members, as well as the interaction between personal attributes and organisational functioning. In our interviews, participants consistently framed their insights at the organisational or team level rather than through individual perspectives. As a result, no data aligned with the constructs in this domain were found. Table 3 provides supporting evidence, including details of constructs and examples of relevant quotes.

**Table 3:** Consolidated Framework Implementation Research (CFIR) constructs identified in participants’ narratives.

Domain	Construct	Example quotes
**1. Intervention** **Characteristic** (point-of-care tools and resources provided by laboratories to support GPs)	**A. Intervention source**	‘Things like educational material that we publish are based on the practice guidelines. But it also takes into account the questions that we are getting asked every day and often we’ll go to conferences and you’ll give a talk about a particular test and then you’ll get a question about how this is different. And so we thought, actually, we need a summary table that compares these two tests so that they can just have a visual and they can see it. If you get a question, you just know, because they are asking the same question, then you know that there is a gap’ [LR 1].
	**B. Evidence strength & quality**	‘We obviously try to be as factually accurate and clear as possible. We have a team of pathologists, genetic counsellors, scientists and also the clinical teams that will provide the general content’ [LR 4].
	**C. Relative advantage**	‘I also think that it’s best not to duplicate things. If there are good things out there, then let’s use those, rather than trying to develop our own’ [LR 8].
	**D. Adaptability**	‘We have developed the reproductive carrier screen course, which we have made available to GPs as well, so that they are able to see the type of information that the patients have access to. We have then made a very succinct summary, with a nice flow chart that essentially adapts the key aspects of the test. But it’s a resource that a requesting doctor can just quickly look at and refer to as a quick point-of-care testing’ [LR 1].
	**E. Trialability**	‘We obviously try to be as factually accurate and clear as possible. We test it *[content of test reports]* with GPs to make sure that they’re able to access, read and understand the materials as intended. So, we will kind of carry out that testing piece and then just get feedback from the GPs in general about if they find it useful’ [LR 4].
	**F. Complexity**	‘Genetics is complex and our reports are complex and that often requires a deep understanding of genomics’ [LR 5]. ‘Explaining what the test is and what we can do is definitely not enough for them [*GPs*] to be guided through assessing a patient for genetic testing. And it’s also about the type of genetic testing, because there are lots of different types and suitability for certain types of testing against what you’re trying to look for as well’ [LR 2].
	**G. Design quality &** **packaging**	‘Certainly, there is a lot of information provided and there are links to counselling services as well, provided by the private healthcare system. So, there are pamphlets and, in practice software-type decision-making tools that link people up to the right information and the right people when ordering those tests’ [LR 3].
	**H. Cost**	‘If we had resources, we could help, but we are just so under the pump that it is very difficult to be able to even develop things that could be sent out’ [LR 8]. ‘Providing that educational component that probably needs to be driven is hard to quantify in terms of the return and so on. So, we may do it on a small scale, like providing educational seminars, supporting workshops and things. But I think for it to be scalable and really have meaningful impact, it needs to be driven at a higher level, on a government level or by academia’ [LR 3].
**2. Outer setting** (The broader healthcare system, government, policy, etc.)	**A. Patient needs &** **Resources**	‘GPs’ understanding, exposure, education and training on genetics really vary, especially depending on when they were educated. I guess for generations of GPs, genetics wasn’t really a thing. Because genetic testing really came into the market probably in the 2000s. So, if you had your medical training well before that, then you really haven’t had that exposure to genetics right in as part of your training’ [LR 4].
	**B. Cosmopolitanism**	‘We work with a lot of clinical geneticists and genetic counsellors, both in public and private’ [LR 4]. ‘They have skills that we don’t have and we have technical skills that they don’t have. So, we complement each other really well’ [LR 2].
	**C. Peer pressure**	‘I think that the private space spends more time doing so *[providing support tools]*, because they at the same time as educating, are competing for a business. Whereas in the public sector, I think the laboratories are very removed from the education of their referrers’ [LR 3].
	**D. External policies &** **incentives**	‘We see a lot of testing requests for things like reproductive carrier screening as it is provided on the Medicare rebate’ [LR 1].
**3. Inner setting** (Laboratories organisations)	**A. Structural characteristics**	‘GPs are able to contact the laboratory to talk to a specialist, genetic pathologist, usually at business hours. Any doctor who wants to discuss either a particular result or has a clinical inquiry is able to contact and telephone us through the helpline and we are able to get back to them, usually straight away if we’re in our offices. But if I’m in a meeting like this, usually within a couple of hours the latest they will have a response from us’ [LR 1].
	**B. Networks & Communications**	‘When we have to work with the clinician on a test like before testing or even after, I will tag them [in-house genetic pathologists] and say, we’re about to issue a report that is quite difficult. So, I need you to be on board with the clinician before we even get to the patient’ [LR 2].
	**C. Culture**	‘Any referral is essentially a referral. It’s a clinical consultation between the requesting doctor and the laboratory and so for them to realize that it’s not just sending the sample and getting the sample tested and getting a result, but knowing that they have specialists working in laboratories, pathologists who are able to take a call, talk about a particular case, advise on what they might want to do next and what the result might mean’ [LR 1].
	**D. Implementation climate**	‘I think mainstreaming of genetic testing is inevitable and is a great thing for the population. So, I think if genetic laboratories can assist in making sure a safe and informed process, then I think we have an obligation to do so’ [LR 7].
	**E. Readiness for** **implementation**	‘Laboratories primarily have a role in ensuring that there is responsible provision of testing. But then, also, I think they’re responsible as well for educating the requesters and having educational resources available both online but also going out and speaking to at conferences’ [LR 1].
**4. Process** (The process by which implementation is accomplished)	**A. Planning**	‘We fund part of a genetic counsellor. So when we have to work with the clinician on the first, like before testing or even after, I will tag them and say, Okay, we’re about to issue a report that is quite difficult. So I need you to be on board with the clinician before we even get to the patient. So we do have access to that’ [LR 2].
	**B. Engaging**	‘If we come to the end of the testing and there’s something really complicated, we will go back to the GP and say, this is going to be really complex to explain. You’re going to need support from a genetic counsellor or a clinical geneticist’ [LR 2].
	**C. Executing**	‘We occasionally get requests that have turned up in our laboratory and our specimen reception team will alert my team that we’ve got a request here from a GP. What should we do about that? And me or someone from my team would probably contact the GP to discuss with them’ [LR 5].
	**D. Reflecting & evaluating**	‘We provide things at the outset and then we’re responsive to inquiries, but I don’t think that we seek feedback often enough, probably from GPs about what it is they need. We say, this is what we’re gonna give you. And I don’t know how well we respond or seek feedback and I think that goes for education as well in a way. So knowing what education is required rather than us determining what education we’d like to deliver is probably an area we could do better’ [LR 3]. ‘We get a lot of feedback like that from our quality assurance. We also work very closely with our clinical genetics department and they would feed back to us anything that they would like to see different or we may ask them’ [LR 8].

### CFIR domain 1: intervention characteristic

This first domain covers the Intervention’s characteristics; i.e., the characteristics of the point-of-care tools and support provided by laboratories for GPs within their practices. Laboratories positioned their support tools as a response to persistent knowledge gaps among GPs. Participants described an iterative development process where interventions were both evidence-informed, drawing on standardised professional guidelines and practice-driven, shaped by recurring questions and routine interactions with GPs (Table 3, Intervention characteristics—Intervention source [LR 1]). Interview participants highly regarded the strength and quality of the provided materials (Table 3, Intervention characteristics— Evidence strength & quality [LR 4]). Adaptability emerged as essential for uptake, with laboratories trying to condense complex genetic concepts into simplified flowcharts, succinct summaries and point-of-care tools tailored to GPs’ time constraints (Table 3, Intervention characteristics—Adaptability [LR 1]). When considering implementation at scale, cost became a salient issue (Table 3, Intervention characteristics—Cost [LR 8]). Participants acknowledged that the cost of providing these supports exceeds the current capacity of laboratories and cannot bridge the implementation gap in primary care, suggesting that broader implementation may require involvement from government, professional bodies or academic institutions (Table 3, Intervention characteristics—Cost [LR 3, LR 8]). A dominant construct identified was the complexity and intricacy of developing materials for GPs, arising from the rapidly evolving field of genomics and the nature of genomics and genetic tests (Table 3, Intervention characteristics—Complexity [LR 2, LR 5]). GPs must navigate multiple test types, costs and indications and interpret nuanced results, often within brief consultations with patients (Table 3, Intervention characteristics—Complexity [LR 2]).

### CFIR domain 2: outer setting

The second domain is the Outer setting: contextual factors that encompass broader system-level influences, such as GPs’ needs and resources, inter-organisational networks, policy contexts and peer pressures, that shape how laboratories develop and implement point-of-care tools and support for GPs. Laboratories tailored their resources and support to account for the variable knowledge, skills and training of GPs, highlighting laboratories’ adaptation to heterogeneity in the outer setting (Table 3, Outer setting—Patient needs & resources [LR 4]). Participants emphasised that the external policies and organisations shaped their practice through professional guidelines and accreditation standards (Table 3, Outer setting— External policies & incentives [LR 1]). These guidelines helped improve the consistency of language used in reporting and educational materials, further ensuring the development of quality, evidence-based materials (Table 3, Outer setting— External policies & incentives [LR 1]). Participants from private laboratories reported being more proactive in developing and disseminating educational resources, which they attributed to both the need to support GPs and patients and competitive pressures (Table 3, Outer setting—Peer pressure [LR 3]). Public laboratories, in contrast, were less directly engaged in GPs’ education. Laboratories reported collaborating with external clinical geneticists, genetic counselors and specialists in the development of educational materials and reporting tools (Table 3, Outer setting—Cosmopolitanism [LR 2, LR 4]).

### CFIR domain 3: inner setting

Turning to the third domain, the Inner setting, we include the organisational structures, culture and implementation climate within laboratories where point-of-care tools and support are implemented. A key well-established structural process that enables timely communication with GPs was direct access to genetic pathologists in laboratories through helplines, direct calls or emails, which support the rapid clarification of test results and clinical queries (Table 3, Inner setting—Structural characteristics [LR 1]). Participants described an organisational culture in which supporting GPs in genetic testing was encouraged, expected and seen as part of laboratory responsibility, with several framing this as an inevitable shift toward mainstreaming genomic testing (Table 3, Inner setting— Implementation climate [LR 7]). The implementation climate further reinforced their role, as participants viewed the mainstreaming of genetic testing as both inevitable and beneficial, creating a professional obligation for laboratories to uphold safe and informed practice (Table 3, Inner setting— Implementation climate [LR 7]). The perceived tension for change indicated a clear readiness for implementation (Table 3, Inner setting—Readiness for implementation [LR 1]). Despite resource constraints, laboratories expressed readiness for implementation by developing educational materials, delivering presentations to inform GPs and maintaining accessible channels for clinical support, such as direct phone lines, emails and follow-up consultations (Table 3, Inner setting—Readiness for implementation [LR 1]). Internal networks and communication systems facilitated coordinated case management, allowing laboratories to proactively identify complex reports and reach out to GPs before results were communicated to patients (Table 3, Inner setting—Networks & communications [LR 2]). A strong culture of clinical responsibility was evident in participants’ transcripts, with laboratories framing each test order as a form of clinical consultation rather than a simple test request (Table 3, Inner setting—Culture [LR 1]). Collectively, these organisational factors shaped how laboratories positioned themselves as essential intermediaries supporting GPs in navigating increasingly complex genetic testing processes.

### CFIR domain 4: implementation process

The activities and strategies used to implement the intervention are the focus of the fourth domain. Participants described planning for the implementation of the intervention as a structured and guideline-driven exercise, beginning with standardisation of practice and clinical pathways to ensure reports and resources align with professional standards (Table 3, Process—Planning [LR 2]). Engaging emerged as a critical strategy, with laboratories proactively involving GPs, genetic counsellors and clinical geneticists when test results were complex. This engagement functioned as both a safety mechanism, ensuring accurate interpretation of results and a means of maintaining continuity of support (Table 3, Process— Engaging [LR 2]). Executing involved active monitoring of test requests by GPs, with laboratory teams contacting referrers for clarification as needed to ensure appropriate tests and prevent misinterpretation (Table 3, Process—Executing [LR 5]). Reflecting and evaluating occurred primarily through feedback passively sought from GPs and clinical genetics departments (Table 3, Process—Reflecting & evaluating [LR 3, LR 8]). While a small number of laboratories described piloting or informally testing resources with some GPs, most participants admitted that, in developing resources, they did not co-design or involve GPs and some didn’t actively seek feedback on whether it worked or not. Instead, laboratories often rely on their own assumptions about GPs’ knowledge gaps or preferences. As a result, the educational materials and tools were perceived as not always aligning with what GPs find useful in their day-to-day practice (Table 3, Process—Reflecting & evaluating [LR 3]). None of the participants reported conducting any kind of formal evaluation of developed resources (Table 3, Process—Reflecting & evaluating [LR 8]).

## Discussion

This is the first study to examine the recent paradigm of laboratory-provided point-of-care tools and resources developed to support GPs in offering and managing genomic tests in Australia from an implementation science perspective. The use of CFIR as a lens enabled a systematic examination of multi-level influences, demonstrating how the integration of genomic point-of-care tools depends not only on content quality but also on organisational culture, networks and system-level pressures. The framework highlighted where implementation efforts are hindered—for example, by lack of planning and formal evaluation, limited feedback from GPs and/or misalignment between laboratory-developed resources and GPs’ actual needs—and revealed the inherent complexity of preparing and delivering these supports. It also illuminated the structural constraints that laboratories cannot address in isolation.

While our findings showed that laboratories have strong internal cultures, networks, communication channels and readiness to implement point-of-care tools and supports, the inherent complexity of genomics remains a dominant barrier. For instance, generating and executing updated and well-designed information was considered resource- and time-intensive, especially when it was necessary to adapt to different GPs’ needs. The rapid pace of genomic advancements can outstrip the updates provided through existing educational resources, creating ongoing challenges for laboratories in preparing materials and supports that remain relevant and appropriate [2]. The language used in genetic test reports has been cited as complex and problematic, with technical genetic terms creating confusion and uncertainty in understanding the content [27]. For example, there has been movement toward standardising the use of the term ‘pathogenic variant’ in place of ‘mutation’ for fragile-x syndrome, using simple language and terminology and encouraging the clarification of ambiguous or outdated genetics terminology [28]. Studies have shown that non-genetic clinicians prefer simpler language, which facilitates understanding and confidence in discussing results [29].

In a nationwide study examining the acceptability and feasibility of RGCS, the use of online modules and decision aids was reported as an effective strategy to support general practitioners in managing the complexity of offering and interpreting tests [30]. This approach was compatible with GPs’ workflows and systems, which improved their engagement with point-of-care tools and materials. Our findings further triangulate with this research, indicating that the compatibility of resources is important for engagement and confidence in those tools and resources [31].

Our findings highlighted a structural misalignment between the kind of support that laboratories design and provide and the actual needs of end-users (GPs). Participants did not report conducting any formal needs assessment for GPs’ education, nor did they engage GPs in co-design processes. This implies a lack of planning and a failure to address key determinants of implementation success, including acceptability, appropriateness and feasibility. Another notable gap is the absence of any formal evaluation beyond internal quality improvement processes. Given that the intervention targets GPs and, secondarily, their patients, this represents a significant oversight. Implementation will remain fragmented until laboratories move from assumption-driven design to co-design, usability testing and structured feedback mechanisms with GPs. In one study in Australia, GPs reported they preferred educational topics in genomics relevant to their practice [32]. This necessitates ensuring that these support and educational materials are interactive to meet the real-world challenges GPs face in ordering and managing genetic tests.

Resources, point-of-care tools and tangible supports have the potential to facilitate the integration of genomics into practice. However, genomic medicine is rapidly expanding and as such, exceeding the capacity of genetic pathology laboratories to meet demand [33]. Participants in this study noted that the substantial cost, time and effort required to produce and maintain educational materials and communication channels with GPs may exceed their capacity. When individual laboratories independently produce educational materials and resources, it increases heterogeneity in quality and availability of support [34]. Furthermore, some GPs may receive comprehensive support, for example, those working in metropolitan and major cities, while others may receive minimal guidance depending on which laboratory they work with; for example, a rural GP may not be able to receive enough support for a test. Providing a repository of genetics and genomics [35] that is appropriate for GPs would reduce duplication, improve consistency and relieve cost pressures for laboratories. Sustainable implementation requires policy, governance and funding mechanisms beyond individual laboratories.

These findings should be interpreted within the broader implementation context of primary care genomics. Integrating genomics into primary care requires a coordinated effort from governments, academic institutions and clinical genetic services, which collectively drive system-wide change through the development of guidelines, workforce education and multidisciplinary care pathways. If primary care is to incorporate genetics effectively, GPs must acquire core competencies, including understanding test indications, interpreting probabilistic results, communicating uncertainty and managing downstream referral pathways. Ideally, these competencies should be embedded across undergraduate and postgraduate medical education to promote an integrated approach to genetic medicine. Further, government involvement through system-level approaches, including coordinated policy leadership, national guidelines and standards, sustainable funding models and accessible specialist support, is therefore essential to support primary care clinicians and to enable the responsible and scalable implementation of genomics in primary care [36–38].

### Limitations and strengths

A strength of this study is that interviews were conducted with representatives from laboratories across all Australian states and territories, in a range of work settings, including both private and public laboratories. This study focused on the Australian context, which may limit the generalisability of findings to other healthcare systems with different structures, funding models or regulations. We acknowledge that variation in health system structures can affect how factors influence implementation outcomes. Nevertheless, by applying a widely adopted framework, CFIR, our aim was to utilise a methodology that could be applied in other contexts to guide implementation. It revealed gaps in the current approach and identified relevant strategies that can promote effective and sustainable implementation. Using CFIR enables consistency across the literature and in devising interventions to improve the effective provision of point-of-care tools and support for integrating genetics in primary care.

Another limitation of this study is that, while the absence of the ‘Individual Characteristics’ domain in the findings reflects the organizational-level focus of the data, it indicates that the analysis does not capture how individual staff beliefs, knowledge or professional roles may influence the development and delivery of point-of-care tools and support. This should be considered when interpreting the findings, as individual-level determinants are recognised as important contributors to implementation success in CFIR-based studies and may operate alongside organisational and system-level factors.

## Conclusion

Our study highlighted the unique position of laboratories to enable the widespread application of genomics in primary care by providing timely and appropriate point-of-care tools and support for GPs. This was achieved through a strong internal culture, clinical responsibility, well-established communication pathways and networks and readiness for implementation, alongside adaptable and evidence-informed materials. Nonetheless, it revealed the fragmented and ad hoc nature of the development of point-of-care tools and resources. Further, the lack of planning, needs assessment and consultation and engagement of GPs has limited its effectiveness. The misalignment between resources developed by laboratories and GPs’ workflow, time pressures and variable genomic literacy was a key finding. Relying on individual laboratories to independently develop tools is unsustainable and contributes to inconsistent quality and access across primary care. Strengthening implementation requires a coordinated national approach, including co-design with GPs, standardised educational resources, shared infrastructure and supportive policy and funding mechanisms. By grounding the findings in an established implementation framework, this study extends the emerging literature on mainstreaming genomics into primary care and provides a structured foundation for designing more effective, scalable implementation efforts.

## Supplementary information

Interview Schedule

Supplementary

## Data Availability

All data generated or analysed during this study are included in this published article.
