# Supplementary material for: The role of pathology laboratories in integrating genetic testing into Australian primary care: an implementation science perspective
Source: Eur J Hum Genet. 2026 Sep 11;34(11):1753–61. doi: 10.1038/s41431-026-02213-8 (PMC13743550; doi:10.1038/s41431-026-02213-8)
Supplement: Supplementary file 1 [file 41431_2026_2213_MOESM1_ESM.docx]

***The Role of Pathology Laboratories in Integrating Genetic Testing into Australian Primary Care: An Implementation Science Perspective***

**Laboratory Interview Schedule**

**Researcher Guide**

- *Cover the topics represented in the 6 questions as appropriate to each informant. Each topic has a set of prompts to use.*
- *Probe on insights into the ways that genomics laboratories support GPs’ desired service outcomes of efficiency, safety, effectiveness, equity, patient-centeredness and timeliness.*

**Semi-structured interview script**

Thank you for agreeing to be interviewed for our study exploring perspectives of Australian genomics testing laboratories on providing resources for primary care.

The interview will take around 30 minutes.

Before we start this interview, can I confirm that you have read the participant information form and signed the consent section?

Do you have any questions about the interviews?

I am now going to switch on the Teams/Zoom record feature. Are you happy for this meeting to be recorded and transcribed by Teams/Zoom? You can keep your camera off if preferred.

We’re going to talk about the kinds of supports and resources your organisation has developed for GPs in relation to genomic diagnostics and screening. We’re particularly interested in preconception and antenatal reproductive genetic carrier screening and also testing for familial hypercholesteremia but we are interested in all genetic testing and screening relevant to general practice in Australia.

**Key Focus 1: Laboratory Roles and Engagement with General Practice**

- Can you describe your current role and responsibilities within the genomics laboratory?
- How do you view the role of genomics laboratories in supporting general practice in Australia?
- What types of genomic tests are now most commonly requested by GPs?

**Key Focus 2: Supporting GP Decision-Making at Point-of-Care**

- Have you developed, or do you consider it within your scope to develop, clinical decision support tools (e.g., point-of-care prompts, risk assessment tools) that integrate into GP software?
- Do you provide, or consider it within your scope to provide, patient-facing materials for GPs to use in consultations?
- What expertise do you draw on when developing these tools and resources?
- How do you ensure these resources are evidence-based, ethically sound, and tailored to diverse populations (e.g., CALD communities, rural/regional areas, patients with disability or chronic illness)?

**Key Focus 3: Supporting Test Ordering, Interpretation and Communication**

- What common challenges or queries do GPs raise about ordering or interpreting genetic tests? How do you address this feedback?
- How does your laboratory ensure that reports contain sufficient and clear information for GPs to confidently explain results to patients?
- Do you provide, or consider it within scope to offer, a consultation service or support line for GPs managing complex genetic results?

**Key Focus 4: Genetic Counselling and Referral Pathways**

- Do your laboratory’s reports or resources guide GPs on when and how to refer patients to genetic counsellors or specialists?
- Does your laboratory provide genetic counselling directly, and are GPs informed when this service is used by their patients?
- Do your resources include or link to patient support organisations? What role should these organisations play after testing?

**Key Focus 5: Enhancing Genomic Literacy and Communication**

- Do you see a role for laboratories in improving the genomic literacy of GPs?
- How do you address inconsistencies in terminology and acronyms across laboratories to avoid confusion in clinical practice?
- Have you received feedback on problematic language in genetic reporting (e.g. “mutation”, “carrier”)? Have you adapted language in your materials in response?

**Key Focus 6: Future Directions for Lab–Primary Care Collaboration**

- How do you envision future collaborations between laboratories and GPs to enhance the use of genomics in primary care?
- What additional resources or support mechanisms would help GPs better integrate genomic testing into their practice, and how could your lab contribute?
- Over the next 5–10 years, how do you foresee the role of genomic testing evolving in Australian primary care?
