# Supplementary material for: The role of pathology laboratories in integrating genetic testing into Australian primary care: an implementation science perspective
Source: Eur J Hum Genet. 2026 Sep 11;34(11):1753–61. doi: 10.1038/s41431-026-02213-8 (PMC13743550; doi:10.1038/s41431-026-02213-8)
Supplement: Supplementary file 2 [file 41431_2026_2213_MOESM2_ESM.docx]

**Title:**

***The Role of Pathology Laboratories in Integrating Genetic Testing into Australian Primary Care: An Implementation Science Perspective***

**Supplementary file 1.** The original and contextual definitions of domains and constructs of the Consolidated Framework for Implementation Research

| **Domain** | **Definition** | **Definition in the context of this study** | **Construct** | **Definition** | **Definition in this context** |
| --- | --- | --- | --- | --- | --- |
| **1. Intervention**  **Characteristic** | The characteristics of the intervention being implemented in a particular organisation | Point-of-care tools and resources provided by laboratories to support GPs | **A. Intervention source** | Perception of key stakeholders about whether the intervention is externally or internally developed. | Point-of-care tools and resources provided by laboratories to support GPs |
|  |  |  | **B. Evidence strength & Quality** | Stakeholders’ perceptions of the quality and validity of evidence supporting the belief that the intervention (published literature, guidelines) will have desired outcomes. | Perception of the strength and validity of tools/support provided by laboratories |
|  |  |  | **C. Relative advantage** | Stakeholders’ perception of the advantage of implementing the intervention versus an alternative solution. | Advantages of providing support and tools for GPs |
|  |  |  | **D. Adaptability** | The degree to which an intervention can be adapted, tailored, refined, or reinvented to meet local needs. | How adaptable are the tools and supports provided by laboratories? |
|  |  |  | **E. Trialability** | The ability to test the intervention on a small scale in the organisation. Piloting allows individuals and groups to build experience and expertise, and time to reflect upon and test the intervention. | Testing or piloting point-of-care tools and supports before scaling up |
|  |  |  | **F. Complexity** | Perceived difficulty of implementation, reflected by duration, scope, radicalness, disruptiveness, centrality, and intricacy and number of steps required to implement. | How easy or complicated is the implementation of tools and resources? |
|  |  |  | **G. Design quality &**  **packaging** | Perceived excellence in how the intervention is bundled, presented, and assembled | Presentation/usability of support materials (e.g., test reports, decision support tools). |
|  |  |  | **H. Cost** | Costs of the intervention and costs associated with implementing that intervention, including investment, supply, and opportunity costs. It is important to differentiate this construct from available resources (part of inner setting, | Cost of providing support/tools |
| **2. Outer setting** | The outer setting includes the economic, political, and social context within which an organisation resides. | The broader healthcare system, government, policy, etc. | **A. Patient needs &**  **Resources** | The extent to which patients’ needs, as well as barriers and facilitators to meet those needs, are accurately known and prioritised by the organisation. | How laboratories take into account the needs of GPs |
|  |  |  | **B. Cosmopolitanism** | The degree to which an organisation is networked with other external organisations | Degree to which laboratories are networked with external organisations (e.g., genetic counsellors, professional bodies, other laboratories). |
|  |  |  | **C. Peer pressure** | Mimetic or competitive pressure to implement an intervention, typically because most or other key peer or competing organisations have already implemented or in pursuit of a competitive edge. (competitors in the market, other hospitals in a network) | How much does the practice of other laboratories impact their function? |
|  |  |  | **D. External policies &**  **incentives** | External strategies to spread interventions, including policy and regulations  (governmental or other central entity), external mandates, recommendations and guidelines | Influence of Medicare, professional bodies and guidelines on laboratory’s services and tools. |
| **3. Inner setting** | The inner setting includes features of structural, political, and cultural contexts through which the implementation process will proceed | Laboratories organisations | **A. Structural characteristics** | The social architecture, age, maturity, and size of an organisation. Social architecture describes how large numbers of people are clustered into smaller groups and differentiated, and how the independent actions of these differentiated groups are coordinated to produce a holistic product or service | The structural characteristics of laboratories, such as their size, staffing, division of roles, etc. |
|  |  |  | **B. Networks & Communications** | The nature and quality of webs of social networks and the nature and quality of formal and informal communications within an organisation. Connections between individuals, units, services, and hierarchies. | The formal and informal channels within laboratories that facilitate information sharing, coordination of case management, and communication with GPs, such as the availability of in-house genetic counsellors. |
|  |  |  | **C. Culture** | Norms, values, and basic assumptions of a given organisation | The shared values, norms, and expectations within laboratories regarding how they perceive providing support for GPs. |
|  |  |  | **D. Implementation climate** | The absorptive capacity for change, shared receptivity of involved individuals to an intervention, and the extent to which use of that intervention will be rewarded, supported, and expected within their organisation. Climate can be assessed through tangible and relatively accessible means such as policies, procedures, and reward systems.  **Relative priority:** Individuals’ shared perception of the importance of the implementation within the organisation.  **Compatibility:** How those align with individuals’ own norms and values  **Tension for change:** The degree to which stakeholders  perceive the current situation as intolerable or needing change.  **Organisational incentives** **and rewards**: Extrinsic incentives such as goal-sharing awards, performance reviews, promotions, and raises in salary  **Goals and feedback:** The degree to which goals are clearly communicated, acted upon, and fed back to staff and alignment of that feedback with goals.  **Learning climate:** leaders express their own fallibility; team members feel that they are essential in the change process. | The degree to which the laboratory environment supports, rewards, and prioritises the development and delivery of point-of-care tools and educational resources for GPs. |
|  |  |  | **E. Readiness for**  **implementation** | Tangible and immediate indicators of organisational commitment to its decision  to implement an intervention, consisting of three subconstructs (leadership engagement, available resources, and access to information and knowledge).  **Leadership engagement:** Commitment, involvement, and accountability of leaders and managers  **Available resources:** The level of resources dedicated for implementation  **Access to information and knowledge:** Ease of access to digestible information and knowledge about the intervention and how to incorporate it into work tasks. Information and knowledge include all sources such as experts, other experienced staff, training, documentation, and computerized information systems. | The laboratory’s preparedness to provide point-of-care tools, including available resources, access to personnel (e.g., genetic pathologists), leadership engagement, etc. |
| **4. Individual**  **Characteristics** | The individuals in the inner involved with the intervention and/or implementation process. | Laboratories’ staff | **A. Knowledge & beliefs about intervention** | Individuals’ attitudes toward and value placed on the intervention, as well as familiarity with facts, truths, and principles related to the intervention. | Staff understanding and attitudes regarding the value, accuracy, and usefulness of point-of-care tools and educational resources for GPs. |
|  |  |  | **B. Self-efficacy** | Individuals’ belief in their own capabilities to execute courses of action to achieve implementation goals  -The more confident an individual feels about his or her ability to make the changes needed to achieve implementation goals, the higher their self-efficacy. | Staff confidence in their ability to provide accurate guidance, develop resources, and support GPs effectively. |
|  |  |  | **C. Individual stage of change** | Characterisation of the phase an individual is in, as he or she progresses toward skilled, enthusiastic, and sustained use of the intervention. | The readiness and willingness of laboratory staff to adopt new practices or tools in supporting GPs. |
|  |  |  | **D. Individual identification with organisation** | A broad construct related to how individuals perceive the organisation and their relationship and degree of commitment to that organisation. These attributes may affect the willingness of staff to fully engage in implementation efforts or use the intervention. | The extent to which staff align with the laboratory’s mission, values, and goals regarding genomic testing support. |
|  |  |  | **E. Personal attributes** | This is a broad construct to include other personal traits. Traits such as tolerance of ambiguity, intellectual ability, motivation, values, competence, capacity, innovativeness, tenure, and learning style have not received adequate attention by implementation researchers. | Individual traits such as capacity, innovativeness, and commitment that influence engagement with the implementation of point-of-care tools. |
| **5. Process** | The implementation process may be an interrelated series of sub-processes that do not necessarily occur sequentially. There are often related processes progressing simultaneously at multiple levels within the organisation. | The process by which implementation is accomplished. | **A. Planning** | The degree to which a scheme or method of behaviour and tasks for implementing an intervention are developed in advance and the quality of those schemes or methods. | How laboratories plan to implement/use support tools. |
|  |  |  | **B. Engaging** | Attracting and involving appropriate individuals in the implementation and use of the intervention through a combined strategy of social marketing, education, role modelling, training, and other similar activities. | Strategies to involve GPs, such as workshops, training, providing online modules, etc. |
|  |  |  | **C. Executing** | Carrying out or accomplishing the implementation according to plan. | How well the support is put into GP’s practice? |
|  |  |  | **D. Reflecting & evaluating** | Carrying out or accomplishing the implementation according to plan. Quantitative and qualitative feedback about the progress and quality of implementation accompanied with regular personal and team debriefing about progress and experience.  Evaluation includes traditional forms of feedback, such as reports, graphs, and qualitative feedback and anecdotal stories of success. Objectives should be specific, measurable, attainable, relevant, and timely (the SMART rubric). | Feedback mechanisms between laboratories and GPs to assess and improve services/tools. |

Reference: (Damschroder et al., 2009)

**References:**

Damschroder, L. J., Aron, D. C., Keith, R. E., Kirsh, S. R., Alexander, J. A., & Lowery, J. C. (2009). Fostering implementation of health services research findings into practice: a consolidated framework for advancing implementation science. *Implementation science*, *4*(1), 50. <https://doi.org/http://www.implementationscience.com/content/4/1/50>
